# Comprehensive computational target fishing approach to identify Xanthorrhizol putative targets

**DOI:** 10.1038/s41598-021-81026-9

**Published:** 2021-01-15

**Authors:** Muhammad Shahid, Ahmad Azfaralariff, Douglas Law, Ahmed Abdulkareem Najm, Siti Aisyah Sanusi, Seng Joe Lim, Yew Hoong Cheah, Shazrul Fazry

**Affiliations:** 1 Department of Food Sciences, Faculty of Science and Technology, Universiti Kebangsaan Malaysia, 43600 Bangi, Selangor Malaysia; 2 Innovative Center for Confectionery Technology (MANIS), Faculty of Science and Technology, Universiti Kebangsaan Malaysia, 43600 Bangi, Selangor Malaysia; 3 ZACH Biotech Depot Private Limited, 43300 Cheras, Selangor Malaysia; 4 Tasik Chini Research Center, Faculty of Science and Technology, Universiti Kebangsaan Malaysia, 43600 Bangi, Selangor Malaysia

**Keywords:** Computational biology and bioinformatics, Drug discovery, Plant sciences

## Abstract

Xanthorrhizol (XNT), is a bioactive compound found in *Curcuma xanthorrhiza* Roxb. This study aimed to determine the potential targets of the XNT via computational target fishing method. This compound obeyed Lipinski’s and Veber’s rules where it has a molecular weight (MW) of 218.37 gmol^-1^, TPSA of 20.23, rotatable bonds (RBN) of 4, hydrogen acceptor and donor ability is 1 respectively. Besides, it also has half-life (HL) values 3.5 h, drug-likeness (DL) value of 0.07, oral bioavailability (OB) of 32.10, and blood–brain barrier permeability (BBB) value of 1.64 indicating its potential as therapeutic drug. Further, 20 potential targets were screened out through PharmMapper and DRAR-CPI servers. Co-expression results derived from GeneMANIA revealed that these targets made connection with a total of 40 genes and have 744 different links. Four genes which were RXRA, RBP4, HSD11B1 and AKR1C1 showed remarkable co-expression and predominantly involved in steroid metabolic process. Furthermore, among these 20 genes, 13 highly expressed genes associated with xenobiotics by cytochrome P450, chemical carcinogenesis and steroid metabolic pathways were identified through gene ontology (GO) and KEGG pathway analysis. In conclusion, XNT is targeting multiple proteins and pathways which may be exploited to shape a network that exerts systematic pharmacological effects.

## Introduction

**Figure 1:**
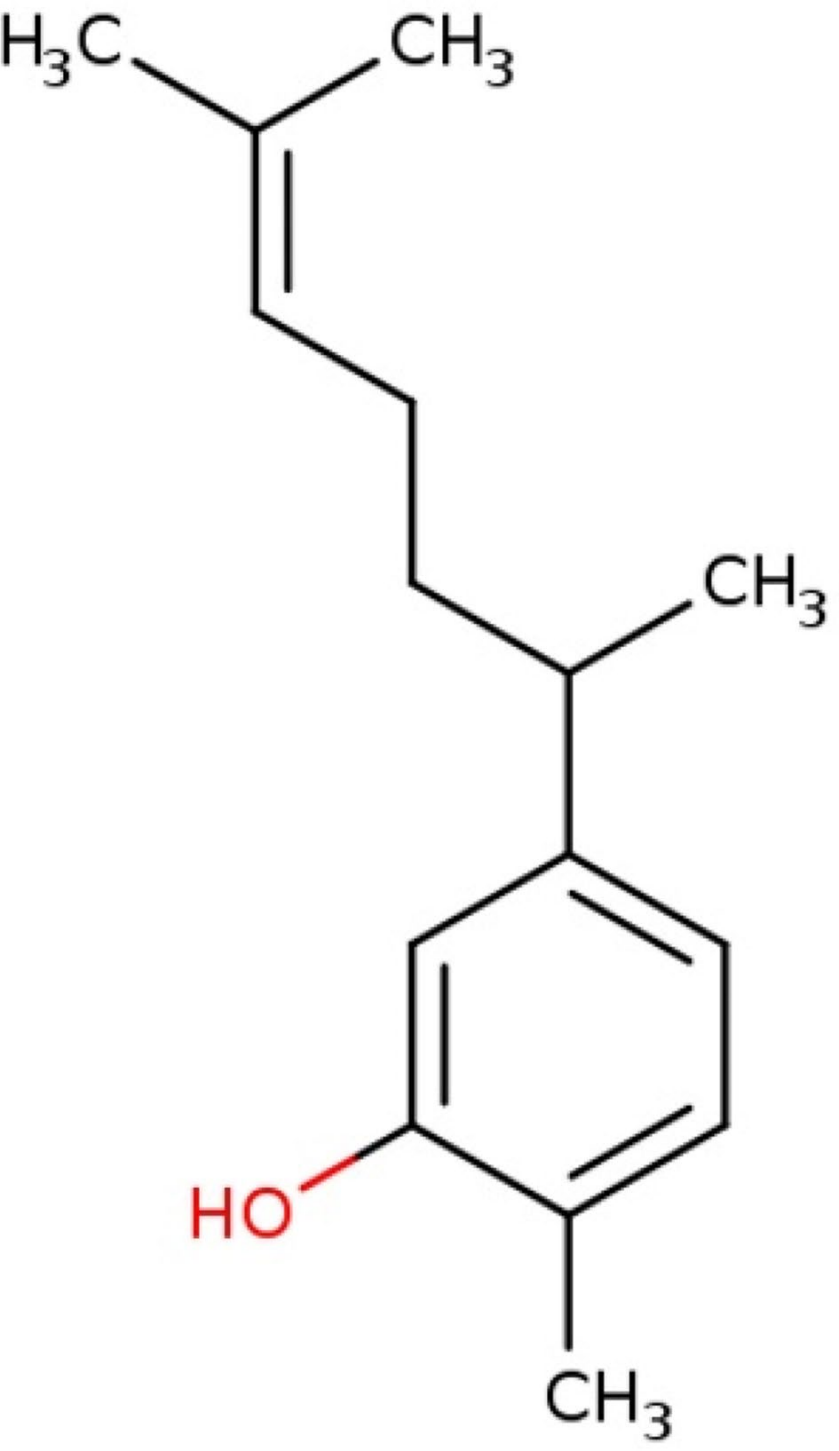
Structure of Xanthorrhizol (XNT).

For centuries, plant bioactive compounds have been widely used for treating a broad spectrum of diseases including cancer^1^. Globally, around 80 percent of the population from developed and developing countries are broadly consumed plant derived drugs^2^. Compounds derived from natural products are considered as promising alternative therapeutic agents due to their potential healing effects^2^. Xanthorrhizol (XNT) (Fig. 1), is a naturally occurring bioactive compound found in *Curcuma xanthorrhiza* Roxb, commonly known as *Java turmeric*^3^. Although, its common name describe its origin from Indonesia, it also widely distributed in Southeast Asia especially in Malaysia, Thailand, Sri Lanka and Philippines^4^. XNT has been reported to be used as hepatoprotective, nephroprotective, antihyperglycemic, antimicrobial, antiplatelets, anti-estrogenic effects and anti-inflammatory^4^. At present most studies are more focus on their putative effects as antioxidant and anticancer agent^5–8^. Due to this it has become an interesting pharmacological compound to be explored further. As the advancement in bioinformatics field, network pharmacology has improved significantly for drug discovery and their design processes^9^. There are various computational target fishing methods such as molecular similarity searching, data mining and machine learning, analysis of bioactivity spectra, protein structure-based and the reverse/inverse docking methods^10–13^. Reverse docking is broadly used powerful tool in which a small molecule (drug) is used to predict the potential binding sites against various macromolecular (proteins) targets. The target fishing approach can facilitate the quick identification of new drug targets, the prediction of the adverse effects, bioactivity and the mode of action of a compound^11^. Despite these advantages, in some cases false positive and false negative results can also expected due to good similarity of inactive compounds with active molecules and limited identification of specific targets of all active compounds respectively^12,14^. Upon developing a novel computational method for target fishing, validation of results with existing one is a fundamental issue^12,15^. Though, in modern drug development, target identification/fishing technology is an emerging approach that has been broadly used^12^.

In this study, reverse docking method was used in drug target fishing to predict potential targets of XNT. The prediction was confirmed through DRAR-CPI server. Various computational target prediction and visualization tools such as DAVID, GeneMANIA, Network analyst, Enrichr and molecular docking were used to identify the underlying targets of XNT. To the best of our knowledge, to date, no comprehensive computational target fishing approach has been used in identifying pharmacological potential of XNT. The aim of this study was to predict the potential target of the XNT via computational target fishing method.

## Results and discussion

### Evaluation of XNT ADME-related properties

For drug development and their clinical succession, ADME (Absorption, Distribution, Metabolism, Excretion) properties of a drug are crucial factors that lead to their approval or rejection^16^. The ADME-related properties of Xanthorrhizol (XNT) were retrieved from the TCMSP and SWISS-ADME servers^17,18^. The TCMSP server contains about all the registered Chinese herbs (499) data with their 29,384 ingredients. It also provides ADME-related information like; molecular weight (MW), human oral bioavailability (OB), Caco-2 permeability (Caco-2), blood–brain barrier (BBB) permeability, drug-likeness (DL), fractional negative accessible surface area (FASA), Topological polar surface area (TPSA), and rotatable bond number (RBN) were presented in Table 1. The results from TCMPS and SWISS-ADME of XNT exhibited that it obeyed the Lipinski’s “rule of five” which states that a molecule should have MW between 180- 500; APLog value (a partition coefficient between water and octanal used to determine hydrophobicity of a molecule) should be less than or equal to five; and hydrogen acceptor and donor value should be less than ten and five respectively^19,20^. It also followed the Veber’s rule which explains that a molecule should have TPSA value (a physiochemical properties represents polarity of a molecule) less than 140 and rotatable bonds in a molecule should be less than 10 where it is considered as good predictor for good oral bioavailability^21^. Moreover, other drug screening criteria, the drug half-life (HL) value is considered as fast-elimination group if HL value is less than four hours (Table 1)^22^. The DL value represents a qualitative concept used in drug design to estimate on how “drug-like” a prospective compound is, which helps to optimize pharmacokinetic and pharmaceutical properties^17^. Remarkably, the Drug likeliness (DL) value of XNT was calculated to be 0.07. The OB value indicating the percentage of orally intake dose of a drug to reach systemic circulation which should be greater than 30%. While for BBB value, it is considered as strong penetrating if the value is greater than 0.3^29^.

**Table 1:** Pharmacological and molecular properties of XNT.

MW	ALogP	H-donor	H-accept	OB (%)	Caco-2	BBB	DL	FASA	TPSA	RBN	HL
218.37	5.07	1	1	32.10	1.72	1.64	0.07	0.32	20.23	4	3.50

### Computational target fishing

Two independent approaches were used to predict chemical-protein interaction namely PharmMapper and DRAR-CPI^23,24^. PharmMapper is an online reverse docking server that quest the chemical-protein targets via pharmacophore mapping approach, while DRAR-CPI is an online server that predicts the adverse drug reaction (ADR) and drug repositioning potential through the chemical-protein interaction (CPI). The PharmMapper and DRAR-CPI produced 249 and 394 match targets respectively by using Z̕-score values (Supplementary Table S1). Generally, in PharmMapper, the large positive value of Z̕-score is considered as significant value while in DRAR-CPI server, the Z̕-score value less than 1 is considered as favorable targets^24,25^. The 20 common targets were screened out from each tool based on the maximum rank of Z̕-score values (Table 2). The OMIM database was explored to identify targets associated human genetic diseases. In Table 2 we summarized all the relevant data of 20 targets included their Z̕-Score, gene name, gene and protein IDs, UniProtKB IDs, OMIM diseases and their inheritance pattern^26,27^.

**Table 2:** Top twenty targets of Xanthorrhizol predicted by PharmMapper and DRAR-CPI serv.

S. no.	Z̕-score	Protein ID	Gene ID	UniProtKB ID	Name	Disease	OMIM ID	Inheritance
1	2.45274	2FKY	*KIF11*	P52732	Kinesin-like protein KIF11	Microcephaly	152,950	AD
2	2.28075	1IE8	*VDR*	P11473	Vitamin D3 receptor	Rickets Type IIA/ Hypo-calcemic Vitamin D-Resistant Rickets	277,440	AR
3	2.0563	19GS	*GSTP1*	P09211	Glutathione S-transferase P	NONE	–	–
4	1.93786	3F0R	*HDAC8*	Q9BY41	Histone deacetylase 8	Cornelia de Lange syndrome 5	300,882	XLD
5	1.92099	3CZR	*HSD11B1*	P28845	Corticosteroid 11-beta-dehydrogenase isozyme 1	Cortisone Reductase Deficiency (CRD)	604,931	AR
6	1.88483	1OIZ	*TTPA*	P49638	Alpha-tocopherol transfer protein	Ataxia with isolated vitamin E deficiency	277,460	AR
7	1.84913	1O6U	*SEC14L2*	O76054	SEC14-like protein 2	NONE	–	–
8	1.70783	2J14	*PPARD*	Q03181	Peroxisome proliferator-activated receptor delta	NONE	–	–
9	1.67823	1ZUC	*PGR*	P06401	Progesterone receptor	Progesterone resistance	264,080	AR
10	1.61551	1PQ2	*CYP2C8*	P10632	Cytochrome P450 2C8	Drug metabolism, altered	618,018	-
11	1.47594	1RBP	*RBP4*	P02753	Retinol-binding protein 4	Retinol-binding protein deficiency- causes night vision problems	180,250	AR/AD
12	1.4032	1MRQ	*AKR1C1*	Q04828	Aldo–keto reductase family 1 member C1	NONE	–	–
13	1.37433	1MA0	*ADH5*	P11766	Alcohol dehydrogenase class-3	NONE	–	–
14	1.19947	1UPW	*NR1H2*	P55055	Oxysterols receptor LXR-beta	NONE	–	–
15	1.17909	1G3M	*SULT1E1*	P49888	Estrogen sulfotransferase	NONE	–	–
16	1.17632	1L8J	*PROCR*	Q9UNN8	Endothelial protein C receptor	NONE	–	–
17	1.06743	1OV4	*SULT2A1*	Q06520	Bile salt sulfotransferase	NONE	–	–
18	1.04296	1OIQ	*CDK2*	P24941	Cyclin-dependent kinase 2	NONE	–	–
19	1.02842	1CG6	*MTAP*	Q13126	S-methyl-5-thioadenosine phosphorylase	Diaphyseal medullary stenosis with malignant fibrous histiocytoma	112,250	AD
20	0.919061	1FBY	*RXRA*	P19793	Retinoic acid receptor RXR-alpha	NONE	–	–

AD; autosomal dominant, AR; autosomal recessive, XLD; X-linked dominant.

### Gene co-expression analysis

**Figure 2:**
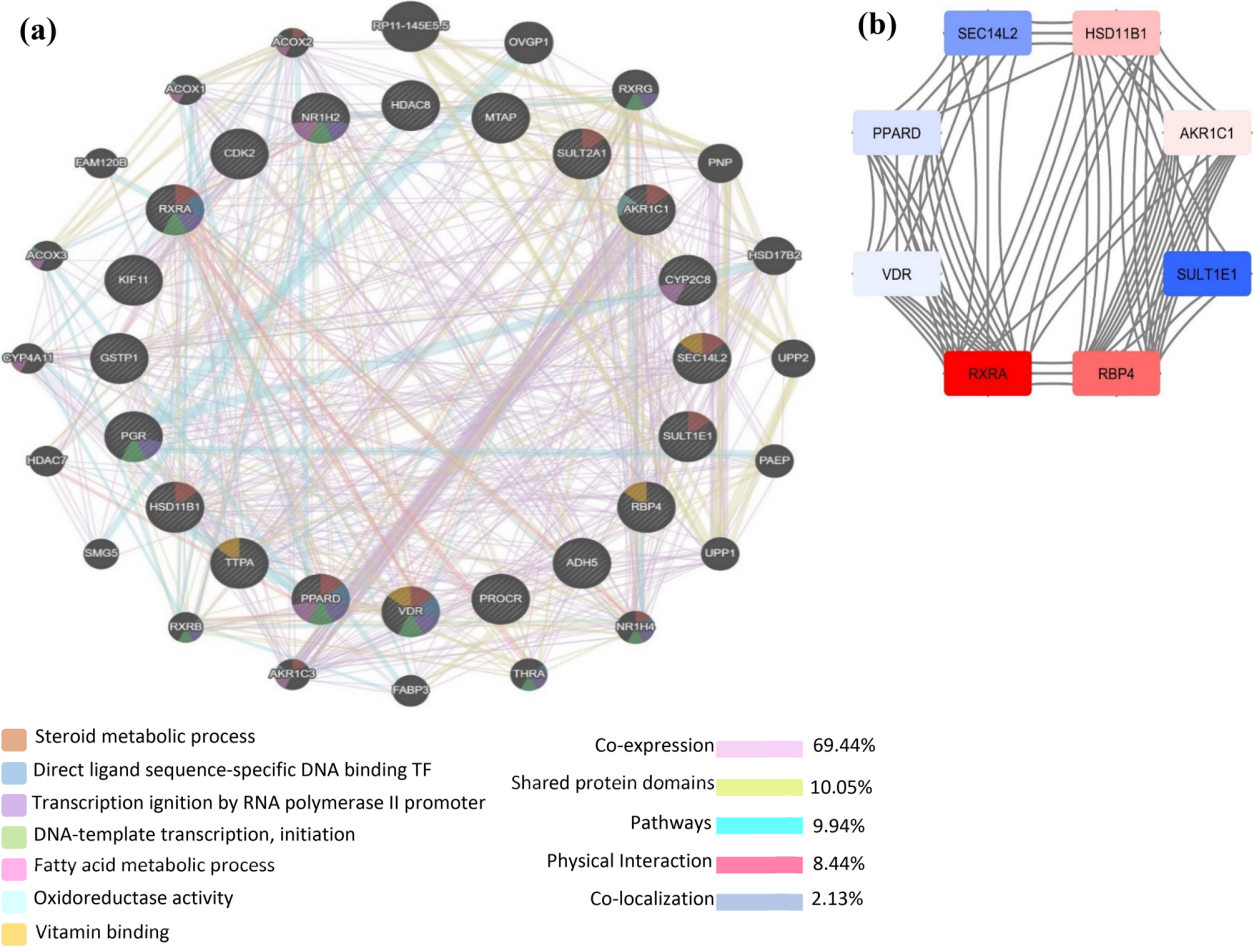
(**a**) Network of co-expression of 20 common targets of XNT constructed with GeneMANIA. The gene are linked to each other by the functional associated networks. Black nodes: gene targets, color nodes represent top expressed genes and their molecular functions filtered on their FDR score, colored lines: represent different interactions. (**b**) The 8 highly co-expressed targets from the GeneMANIA network identified via cytoscape.

To predict co-expression of the genes, GeneMANIA web interface was used^28^. The results of the analysis using GeneMANIA show that these 20 targets have a strong correlation with the other 20 genes. A total of 744 different links have been predicted to build a network that connects these 40 genes (Fig. 2a). The constructed network exhibited the 69.44% similar co-expression characteristics and 10.05% shared the same protein domains. In addition to co-expression and protein domain characteristics, Fig. 2a displayed the other outputs such as colocalization (2.13%), pathways (9.94%), and physical interactions (8.44%) of the twenty targets. The GeneMANIA network also depicted the molecular functions of the top ranked targets that filtered on their FDR score (False Discovery Rate). FDR (≤ 0.00005, Supplementary Table S2) is employed in multiple-comparison testing to screen out differential gene expression by adjusting the raw *p*-value to eliminate false positive rate in data prediction^29–32^. In GeneMania, the GO categories were reported on FDR corrected hypergeometric test for enrichment. The network illustrated that these genes are involve in steroid metabolic process, direct ligand regulated sequence-specific DNA binding transcription activity, DNA-templated transcription initiation process, transcription initiation from RNA polymerase II promoter, fatty acid metabolic process and vitamin binding process (Fig. 2a).

Through cytoscape tool, a network of eight highly co-expressed genes was constructed based on their more connected nodes as showed in Fig. 2b. Among the eight highly co-expressed genes network, four genes which were *RXRA, RBP4, HSD11B1* and *AKR1C1* showed remarkable co-expression and predominantly involved in steroid metabolic process. These results suggested that XNT might be proved as potent compound that exerts its potential on the steroid metabolic process candidates.

### KEGG, GO and network analysis

In this study, for Gene Ontology and pathway analyses, DAVID version 6.8 was used to assimilate biological data such as cell phenotypes, molecular pathways and regulatory networks, which assist in prediction and interpretation of drug and their target bioactivities^12,33,34^. DAVID is an online freely accessible tool that provides a comprehensive biological information of large list of genes, especially gene functions and pathways^34^. In addition, to validate the results of DAVID, two more tools namely Network Analyst and Enrichr were employed^35,36^. Both these tools (Network Analyst and Enrichr) were also web-based complex met-analysis and visualization tools broadly used for gene expression, functional analysis and transcriptional factor analysis^35,36^.

**Figure 3:**
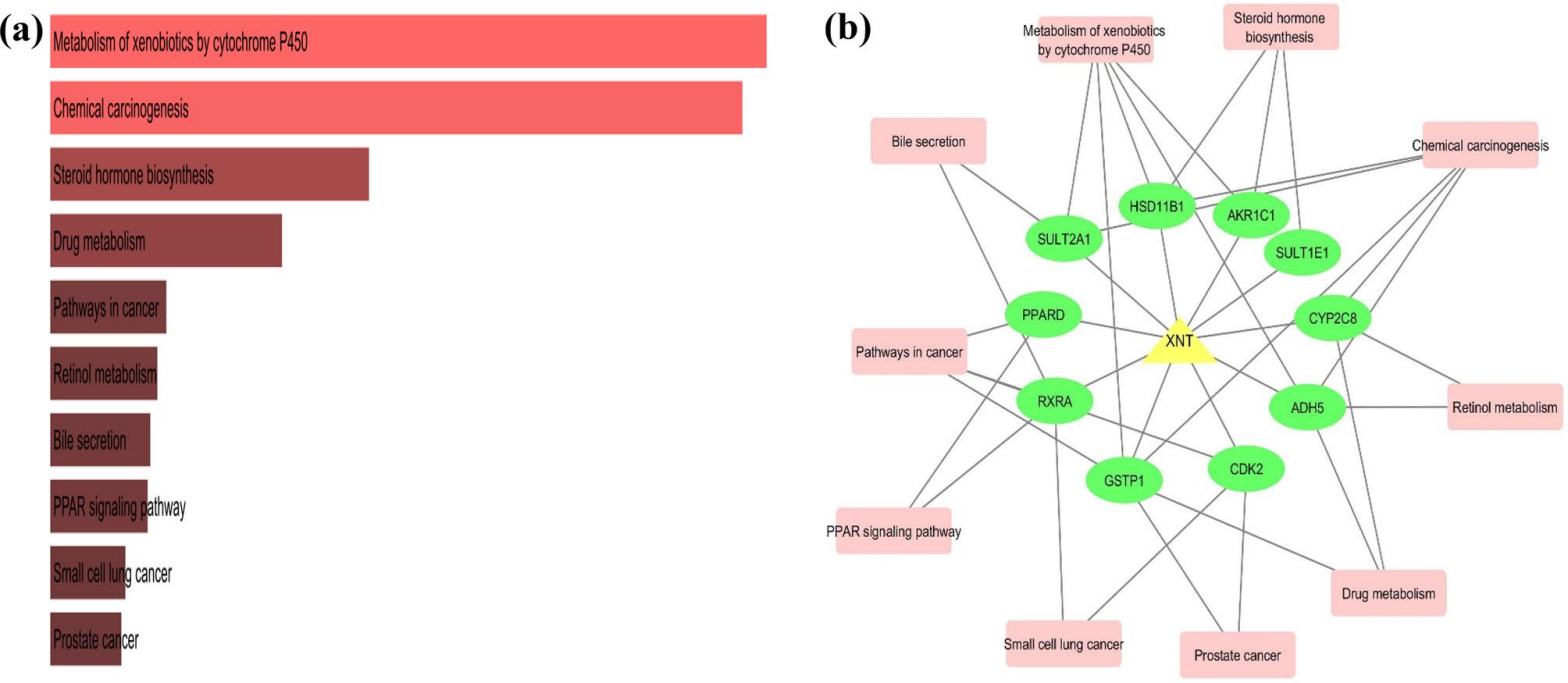
(**a**) Bar plot of KEGG analysis showed top ten enriched pathways**.** (**b**) Drug-target-pathway network constructed by cytoscape illustrated top ten pathways and their associated genes.

In total, 13 KEGG pathways were enriched with the twenty targets of XNT. Figure 3a showed top ten significantly enriched KEGG pathways. A drug-target-pathway network was constructed by cytoscape that illustrated 10 genes remarkably associated with the top ten KEGG pathways. Interestingly, most of the candidates from the network were involved in three predominant pathways which were metabolism of cytochrome P450, chemical carcinogenesis and steroid hormone biosynthesis pathways (Fig. 3b). The KEGG enrichment analysis highlighted the targets and pathways where XNT exert its potential effectively. The cytochrome P450 is a family of enzymatic proteins that play remarkable role in the detoxification of xenobiotics, metabolism of carcinogens, steroids and retinol metabolic pathways^37,38^. The previous studies have remarkably been shown that XNT is a potent anticarcinogenic agent that suppress carcinogenesis via incorporating in apoptotic pathways, anti-inflammatory, anti-oxidant properties and by cell cycle arrest pathways^3,5^. The growth inhibitory effect of XNT has also been elaborated by several studies which were on colon, tongue and esophageal cancer^4,39,40^. In addition, it also showed the ability as an antiproliferative and inhibitor when tested on human hepatoma and breast cancer cells^5,6,41^. A recent study documented the tumor suppressive role of XNT on the prostrate carcinoma cells which revealed that XNT exerts its antiproliferatory impact by inducing G1 cycle arrest^42^. However, the precise mechanism of influence on cell cycle regulation of XNT and underlying molecular targets still remain to be discovered. We have also run similar analysis using WikiPathways algorithm and obtained similar result confidence, comparable to KEGG analysis (Supplementary Table S3).

**Figure 4:**
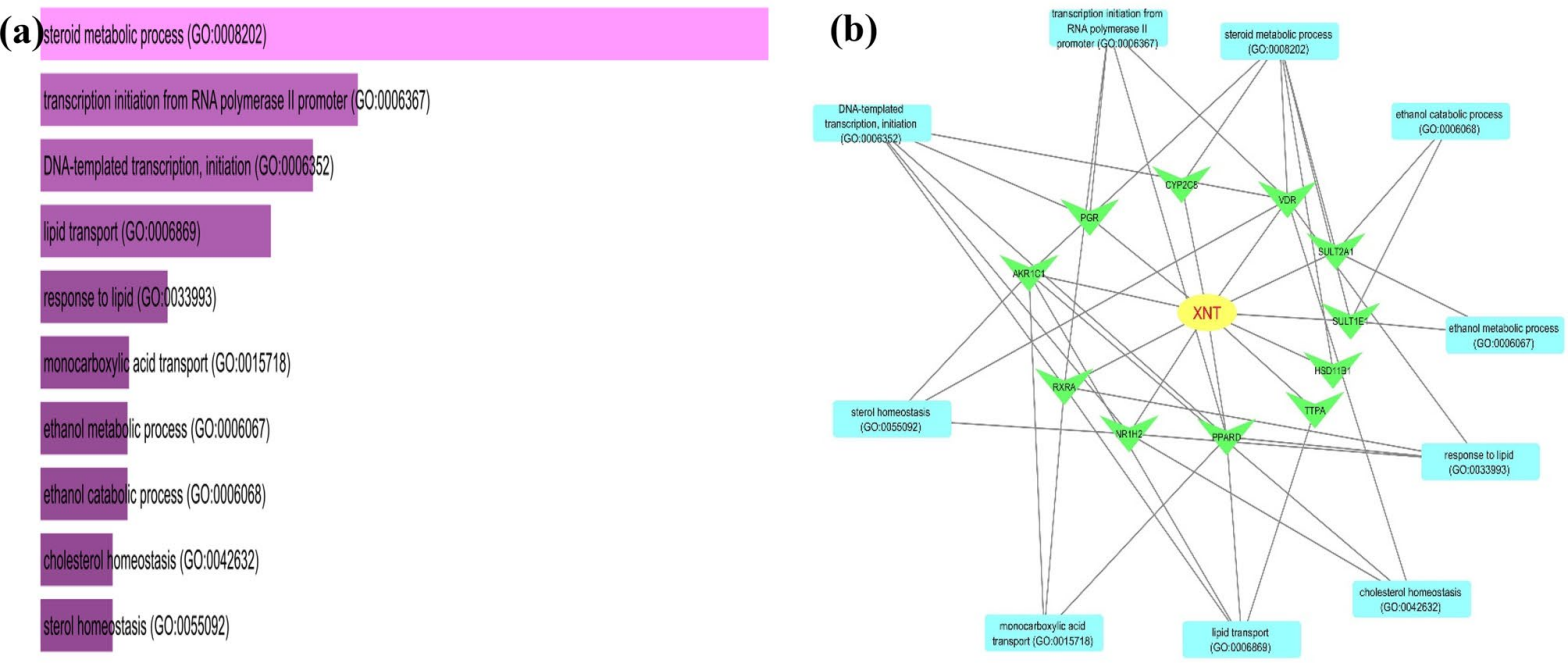
(**a**) Bar graph of top ten enriched biological processes (**b**) Drug-target-biological process network constructed by cytoscape highlighted top ten BP and their linked genes.

**Figure 5:**
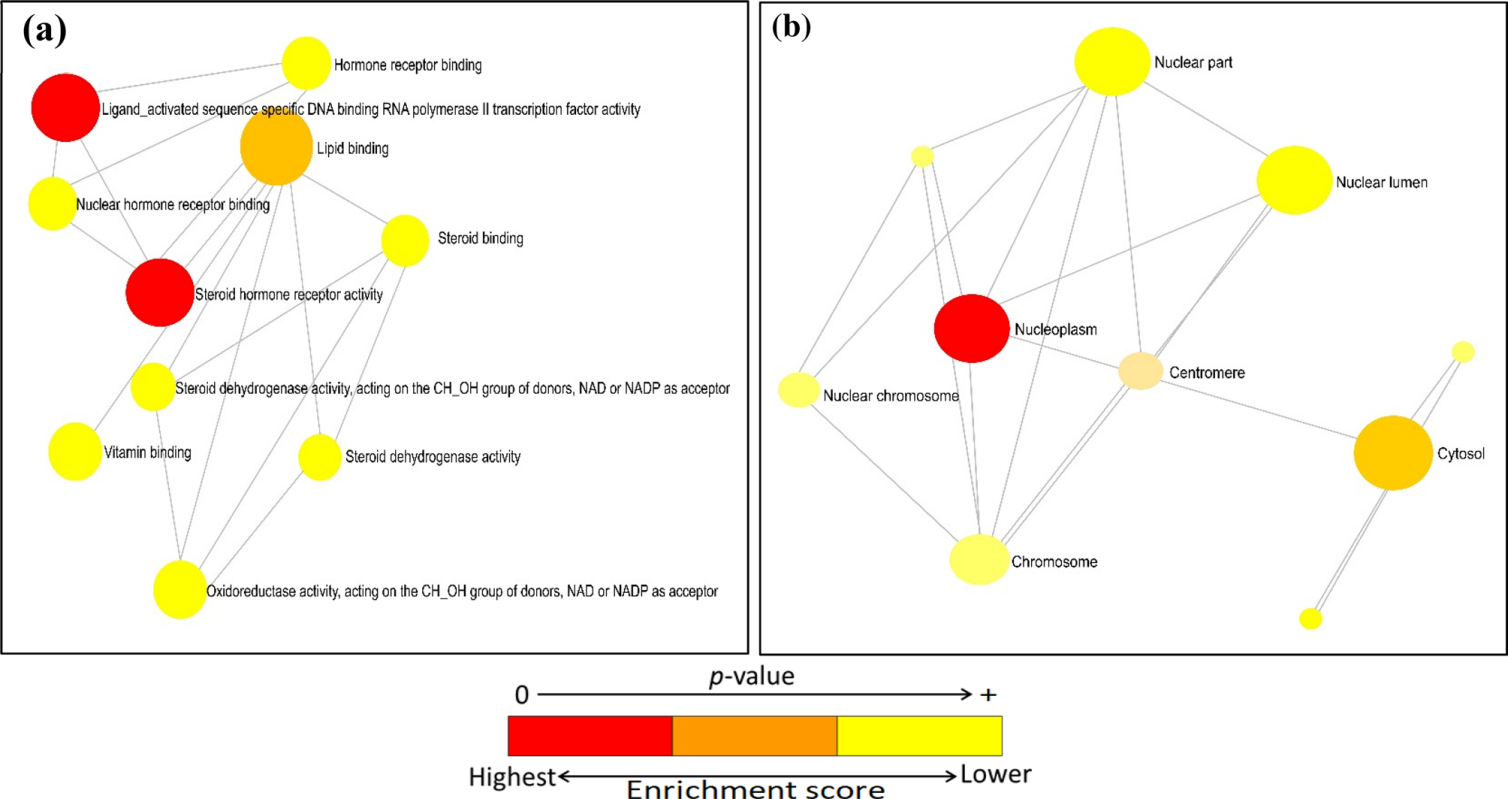
Networks of top ten enriched molecular functions (**a**) and cellular components (**b**) constructed by Network Analyst. In both networks, nodes are color shaded according to their enrichment score (red > orange > yellow).

Furthermore, GO analysis is a useful tool to discover the biological process, cellular component and molecular functions of the genes^43^. In total, 420 biological processes (BP), 78 molecular functions (MF) and 45 cellular components (CC) of the twenty selected genes were identified (Supplementary Table S4). Based on the P-value less than 0.05, 159 BP were filtered from the 420 BP. The functional enrichment analysis of the top ten BP demonstrated that most of the targets were correlated with the regulation of steroid metabolic processes, transcription regulation via RNA polymerase II promoter, DNA-templated transcription initiation, lipid transport and response processes and so on, as showed in Fig. 4a. In addition, a drug-target-biological process network was also constructed by cytoscape which illustrated that 11 genes were predominantly enriched with the top ten BP (Fig. 4b). Subsequently, of the total 78 molecular functions, 37 filtered based on the P-value (< 0.05). The top ten functionally enriched MF are presented in Fig. 5a. Among the top ten molecular functions, two MF such as RNA polymerase II transcription factor activity and steroid hormone receptor activity were highly expressed by our 20 target genes (Fig. 5a). Similarly, the cellular component widely distributed in cytosol, intra and extracellular organelle parts, and nucleoplasm (Fig. 5b).

By comparing the top ten biological processes and pathways enrichment data, seven genes found to be common which were RXRA, PPARD, SULT2A1, HSD11B1, AKR1C1, SULT1E1, and CYP2C8. These genes were mostly involved in steroid metabolic process, metabolism of xenobiotic by cytochrome P450, chemical carcinogenesis/cancer pathways and retinol metabolism. The previous studies have also been revealed the XNT role in the detoxification of xenobiotics by cytochrome P450 and anti-carcinogenic potential but a little work on the incorporation of XNT in steroid metabolic processes. There is still no study been performed which revealed the role of XNT in retinol metabolism. In addition to previously reported functions of XNT, this computational approach uncover and highlighted the targets and pathways where XNT exerts its potential effectively.

### PPI network and docking study

**Figure 6:**
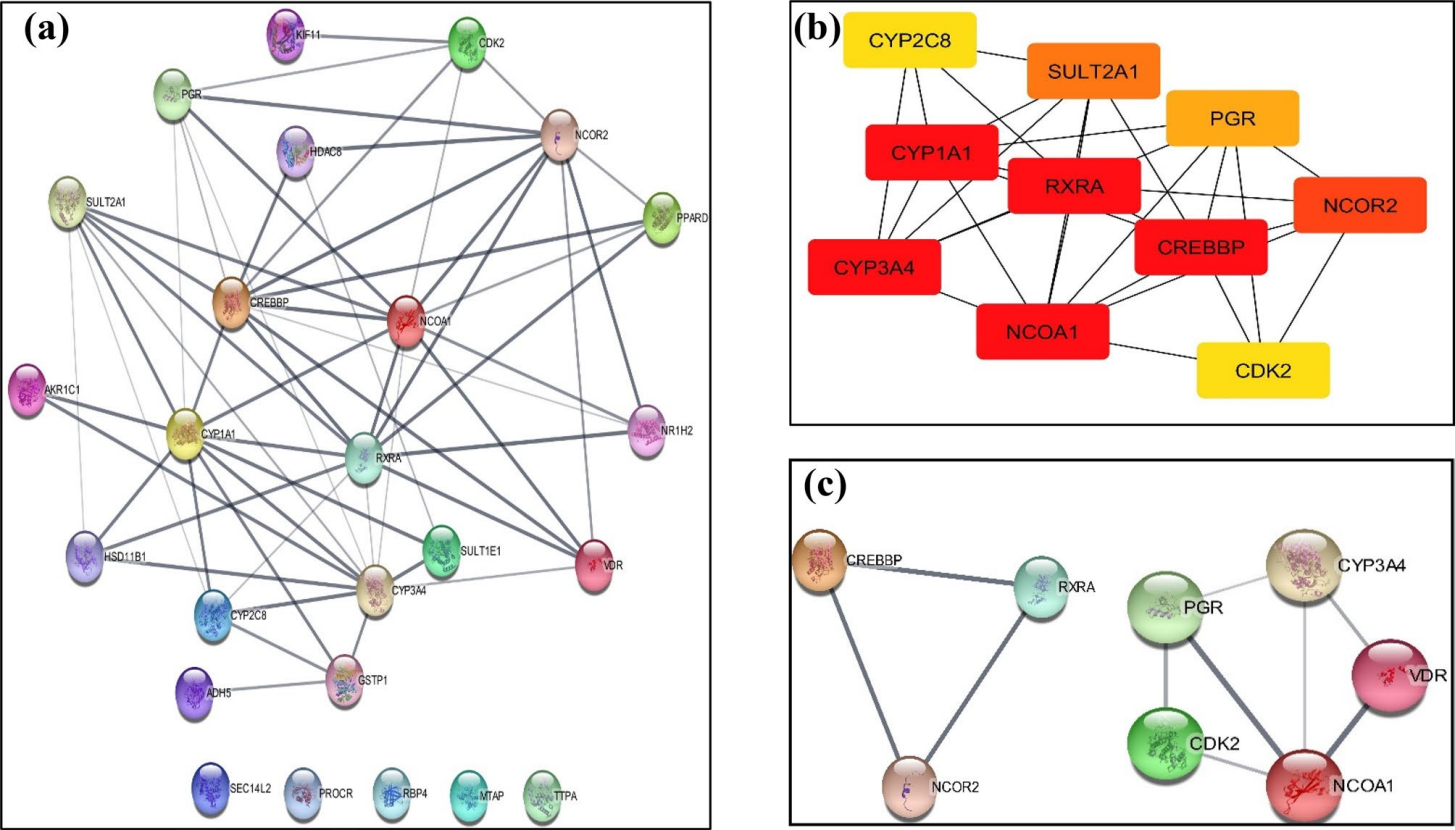
(**a**) PPI network constructed with STRING (The bold line represents direct interaction while the thin line represents indirect interaction), (**b**) Network of hub genes, and (**c**) two modules predicted by cytoHubba and MCODE plugins of cytoscape.

The constructed PPI network reflected the direct (physical) and indirect (functional) association of the XNT targets (Fig. 6a). The cytoHubba plugin of cytoscape was used to identify hub genes from the PPI network. Ten hub genes were identified by cytoHubba which included; *RXRA, CYP1A1, CYP3A4, CYP2C8, CREBBP, NCOA1, NCOR2 CDK2, SULT2A1* and *PGR* (Fig. 6b). The darker color, represent the more important it was; therefore, it suggested that these are the top targets of XNT that plays significant role in biological processes. Similarly, MCODE plugin was run to determine the modules of PPI network. Two modules were found, one module consisted of 5 candidates (*PGR, VDR, CDK2, CYP3A4* and *NCOA1*) and the second module contained 3 i.e. *RXRA, NCOA2* and *CREBBP* (Fig. 6c).

**Figure 7:**
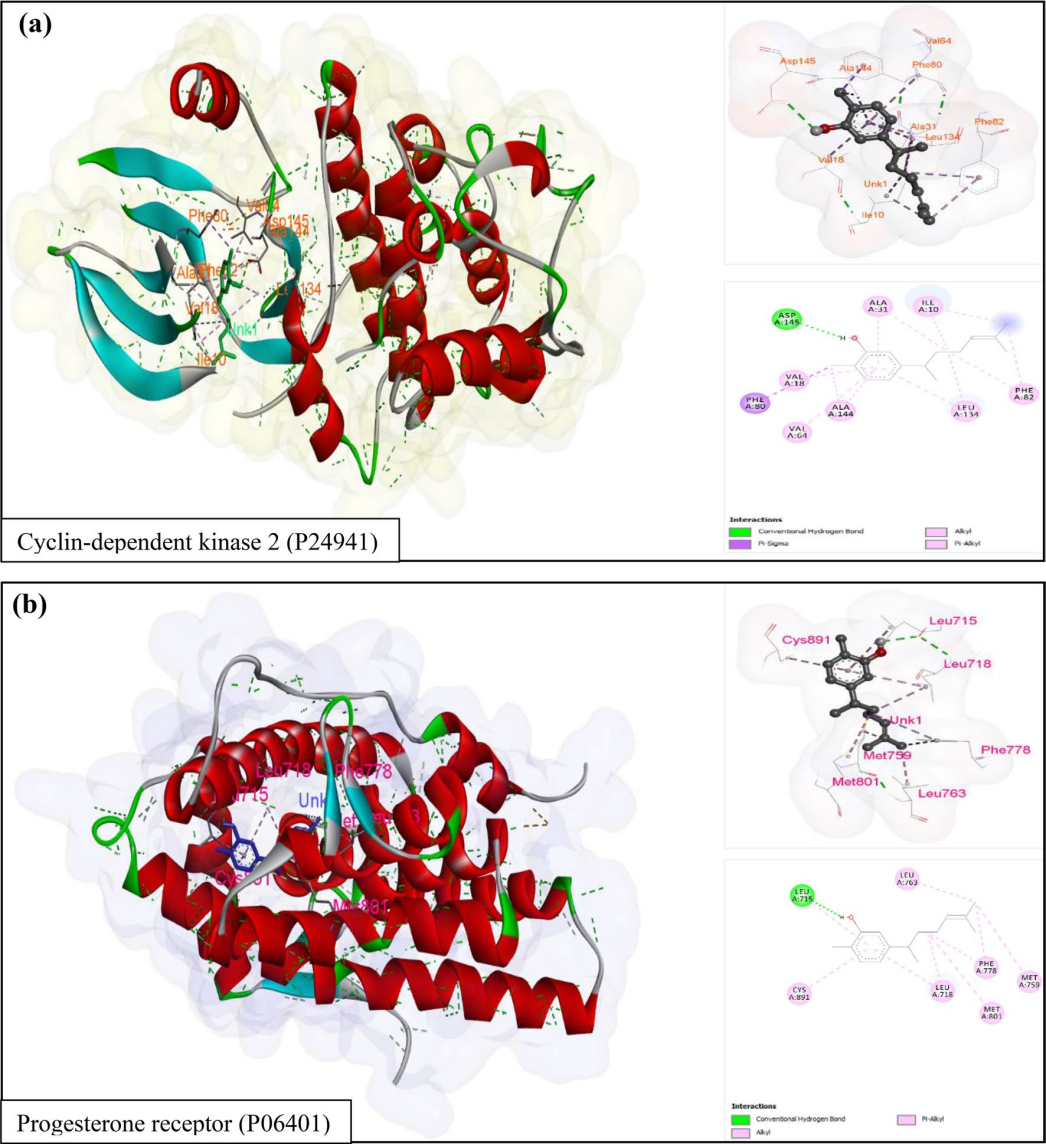
Docking results of XNT with (**a**) Cyclin-dependent kinase 2 (P24941), and (**b**) Progesterone receptor (P06401) selected proteins. Also showed their interacted amino acid residue types and numbers.

The docking results demonstrated the normal binding of ligand with their target proteins, thus, confirmed the interaction that predicted by both PharmMapper and DRAR-CPI servers. Figure 7 showed docking results i.e. interaction visualization and ligand binding sites of the selected proteins (protein IDs; P24941and P06401).

The previous research data extensively explored the XNT therapeutic potential and demonstrated its way of action as hepatoprotective, nephroprotective, antihyperglycemic, antimicrobial, antiplatelet, anti-inflammatory, antioxidant and anticancer agent^44–49^. It is believed that XNT regulates mitogen-activated protein kinase (MAPK) and nuclear factor kappa B (NF-kB) and act as antimicrobial agent. The anti-inflammatory action of XNT via down-regulate the activity of cyclooxygenase-2 (COX-2) and inducible nitric oxide synthase (iNOS), and by inhibition of cytokine interleukin-6 (IL-6) and tumor necrosis factor-α (TNF-α)^45^. Several in vivo studies have been revealed XNT can reduce the insulin, glucose, free fatty acid, and triglyceride levels indicating its antihyperglycemic effects^47^. XNT exhibits antioxidant effects through the regulation of cytochrome p450 enzyme system. Recent studies extensively explored the anticancer potential of XNT against oral cancer, esophageal cancer, skin cancer, breast cancer, colon cancer, liver cancer, ovarian cancer and lung cancer^4,8,49,50^.

Consistent with the previous data, results of the present study also revealed that XNT is incorporated in steroid metabolic process, metabolism of xenobiotic by cytochrome P450, chemical carcinogenesis/cancer pathways and a newly predicted retinol metabolism pathway. Besides previously reported candidates (i.e. MAPK, NF-kB IL-6 TNF-α) that regulated by XNT, this study also indicated that XNT have some more targets prominently are RXRA, PPARD, SULT2A1, HSD11B1, AKR1C1, SULT1E1, and CYP2C8. Although, XNT has multiple application but more in vivo experimental work, pharmacological response via pharmacodynamic approaches, drug concentration at the site of action, and clinical studies are still to be required in order to establish XNT as a standard drug.

## Methods

### Evaluation of XNT ADME-related properties

The ADME (Absorption, Distribution, Metabolism, Excretion) properties were identified by using the Traditional Chinese Medicine Systems Pharmacology Database and Analysis Platform (TCMSP) server (http://www.tcmspw.com/tcmsp.php) and SWISSADME web tool (http://www.swissadme.ch/)^17,18^. In this study, the ADME properties of XNT were identified by using TCMSP server and confirmed through SWISSADME web tool. The chemical structure of XNT was drawn by an online chemistry tool, Chem-Space (https://chem-space.com/) (Fig. 1).

### Computational target fishing

Two computational tools were used for target fishing namely PharmMapper (http://www.lilab-ecust.cn/pharmmapper/) and DRAR-CPI (https://cpi.bio-x.cn/drar/)^23,24^. Before conducting, computational target fishing, the molecular file (SDF) of XNT was downloaded from PubChem drug databank (PubChem CID: 93,135) (https://pubchem.ncbi.nlm.nih.gov/)^51^. The SDF file was uploaded to the PharmMapper server and all targets option (v2010, 7302) were selected while leaving all other parameter set as default^25^. Similarly, the file was also uploaded to DRAR-CPI server and all options were set as default. The overlapping potentially interacting protein targets were chosen base of their maximum rank of Z̕-score from both servers for further investigation for instance Online Mendelian Inheritance in Man (OMIM; https://omim.org/) was used for human genetic disorders prediction, GeneMANIA for co-expression of the genes, DAVID, Network Analyst and Enrichr for GO and KEGG enrichment analysis, STRING for Hub targets and cluster network prediction, PyRx and Discovery studio software’s for molecular docking of XNT and target proteins.

### Gene co-expression analysis

For gene functional analysis, especially to check the co-expression of genes, GeneMANIA (https://genemania.org/) web tool was used^28^. Currently, it supports nine organisms and depicts seven different unique categories i.e. co-expression, co-localization, attribute, genetic interaction, pathway, physical interaction and shared protein domains^28^. Firstly, the list of twenty targets with their protein IDs was searched on UniProtKB (https://www.uniprot.org/uniprot/) database to retrieve their gene IDs^27^. Then, the prepared list of 20 genes was submitted on the GeneMANIA after selecting Homo sapiens from nine available organisms. Moreover, to identify highly co-expressed genes from the constructed GeneMANIA network, cytoscape version 3.7.2 (https://cytoscape.org/) was utilized^52^.

### GO function, KEGG and network analysis

Gene ontology (GO) and Kyoto Encyclopedia of Genes and Genomes (KEGG) pathway of 20 selected targets were analyzed with DAVID (The Database for Annotation, Visualization and integrated Discovery) version 6.8 (https://david.ncifcrf.gov/)^34,53^. The list was submitted on the DAVID server and set background option as *Homo sapiens*. In addition, to validate DAVID outcomes two more biological tools namely Network analyst (https://www.networkanalyst.ca/) and Enrichr (https://amp.pharm.mssm.edu/Enrichr/) were used^35,36^. In order to identify the complex relationship between compound, targets, pathways and biological processes, cytoscape software was used to construct and analyze three-layer networks.

### Protein–protein interaction (PPI) and molecular docking study

A protein–protein interaction (PPI) network of the twenty selected targets of XNT was directly constructed via cytoscape integrated STRING database (https://string-db.org/) with cutoff score 0.4 and maximum additional interaction of 5^52,54,55^. Subsequently, on the established PPI network, CytoHubba and MCODE (the Molecular Complex Detection) plugins of cystoscope were applied. The cytoHubba plugin constructed the network based on the highest degree of neighborhood algorithm from the PPI network. The values for MCODE analysis were set as degree cutoff score; 2, node cutoff score; 0.2, K-core value; 2 and maximum depth; 100.

Furthermore, to validate the ligand–protein interaction which was exhibited by PharmMapper and DRAR-CPI server, molecular docking study was performed. The ligand (XNT) SDF file was acquired from PubChem (CID: 93,135) and PDB files of two randomly selected proteins from the MCODE predicted results were retrieved from Protein Data Bank (https://www.rcsb.org)^56^. The ligand and protein files were prepared through *BIOVIA* Discovery Studio Visualizer version 20.1.0 software and Autodock Vina docking was executed by using PyRx biological software v 0.8 (https://pyrx.sourceforge.io/)^57,58^. The visualization of docking results was performed by Discovery Studio Visualizer.

## Conclusion

The computational target fishing is an emerging approach which assist in drug discovery, design, biomarkers detection, and to investigate the drug-disease relationship. In the present study, the ADME properties of XNT was evaluated by TCMSP and SWISSADME, and potential targets identified by both PharmMapper and DRAR-CPI were projected for further evaluation. The results showed that XNT may be a good drug candidate, and 20 potential interacting targets were identified, of which 13 were highly associated with various pharmacological activities. In addition, GO and pathway analysis was performed and drug-target association networks were constructed. These results demonstrated that XNT has multiple targets and therapeutic potential to regulate crucial biological pathways predominantly metabolism of xenobiotics by cytochrome P450, chemical carcinogenesis and steroid metabolic pathway. The present study provided the comprehensive in silico-based information of XNT possible pharmacological effects that can be used in further experimental research studies to validate their effect.

## Supplementary Information

Supplementary Information 1.

Supplementary Information 2.

Supplementary Information 3.

Supplementary Information 4.

## Abbreviations

ADME: Absorption, distribution, metabolism, excretion
ADR: Adverse drug reaction
BBB: Blood brain barrier
BP: Biological processes
CC: Cellular components
CPI: Chemical-protein interaction
DL: Drug-likeness
DAVID: Database for annotation, visualization and integrated discovery
DS: Discovery studio
FDR: False discovery rate
FASA: Fractional negative accessible surface area
GO: Gene ontology
GeneMANIA: Multiple Association Network Integration Algorithm
HL: Half-life
KEGG: Kyoto encyclopedia of genes and genomes
MW: Molecular weight
MF: Molecular functions
MCODE: Molecular complex detection
OB: Oral bioavailability
OMIM: Online mendelian inheritance in man
PPI: Protein–protein interaction
RBN: Rotatable bond number
STRING: Search tool for the retrieval of interacting genes/proteins
TPSA: Topological polar surface area
TCMSP: Traditional Chinese medicine systems pharmacology
UniProtKB: The universal protein resource knowledgebase
XNT: Xanthorrhizol

## References

[1] Majolo, F., Delwing, L. K. d. O. B., Marmitt, D. J., Bustamante-Filho, I. C. & Goettert, M. I. Medicinal plants and bioactive natural compounds for cancer treatment: important advances for drug discovery. *Phytochem. Lett.* **31**, 196–207 (2019).

[2] Ekor M (2014). The growing use of herbal medicines: issues relating to adverse reactions and challenges in monitoring safety. Front. Pharmacol..

[3] Kim MB, Kim C, Song Y, Hwang JK (2014). Antihyperglycemic and anti-inflammatory effects of standardized curcuma xanthorrhiza roxb: extract and its active compound xanthorrhizol in high-fat diet-induced obese mice. Evid. Based Complement. Altern. Med. eCAM..

[4] Oon SF (2015). Xanthorrhizol: a review of its pharmacological activities and anticancer properties. Cancer Cell Int..

[5] Handayani T, Sakinah S, Nallappan M, Pihie AHL (2007). Regulation of p53-, Bcl-2-and caspase-dependent signaling pathway in xanthorrhizol-induced apoptosis of HepG2 hepatoma cells. Anticancer Res..

[6] Tee, T. T., Cheah, Y. H., Meenakshii, N., Mohd Sharom, M. Y. & Azimahtol Hawariah, L. P. Xanthorrhizol induced DNA fragmentation in HepG2 cells involving Bcl-2 family proteins. *Biochem. Biophys. Res. Commun.* **420**, 834–838. 10.1016/j.bbrc.2012.03.083 (2012). 10.1016/j.bbrc.2012.03.083 22465013

[7] Jantan I, Saputri FC, Qaisar MN, Buang F (2012). Correlation between chemical composition of Curcuma domestica and Curcuma xanthorrhiza and their antioxidant effect on human low-density lipoprotein oxidation. Evid. Based Complement. Altern. Med..

[8] Cheah YH (2008). Antiproliferative property and apoptotic effect of xanthorrhizol on MDA-MB-231 breast cancer cells. Anticancer Res..

[9] Boezio B, Audouze K, Ducrot P, Taboureau O (2017). Network-based approaches in pharmacology. Mol. Inform..

[10] Ning K, Zhao X, Poetsch A, Chen WH, Yang J (2017). Computational molecular networks and network pharmacology. Biomed. Res. Int..

[11] Wang L, Xie X-Q (2014). Computational target fishing: What should chemogenomics researchers expect for the future of in silico drug design and discovery?. Future Med. Chem..

[12] Cereto-Massagué A (2015). Tools for in silico target fishing. Methods.

[13] Sydow D (2019). Advances and challenges in computational target prediction. J. Chem. Inf. Model..

[14] Hawkins PC, Skillman AG, Nicholls A (2007). Comparison of shape-matching and docking as virtual screening tools. J. Med. Chem..

[15] Bender A (2007). Chemogenomic data analysis: prediction of small-molecule targets and the advent of biological fingerprints. Combin. Chem. High Throughput Screen..

[16] Lucas AJ, Sproston JL, Barton P, Riley RJ (2019). Estimating human ADME properties, pharmacokinetic parameters and likely clinical dose in drug discovery. Expert Opin. Drug Discov..

[17] Ru J (2014). TCMSP: a database of systems pharmacology for drug discovery from herbal medicines. J. Cheminform..

[18] Daina A, Michielin O, Zoete V (2017). SwissADME: a free web tool to evaluate pharmacokinetics, drug-likeness and medicinal chemistry friendliness of small molecules. Sci. Rep..

[19] Lipinski CA, Lombardo F, Dominy BW, Feeney PJ (1997). Experimental and computational approaches to estimate solubility and permeability in drug discovery and development settings. Adv. Drug Deliv. Rev..

[20] Viswanadhan VN, Ghose AK, Revankar GR, Robins RK (1989). Atomic physicochemical parameters for three dimensional structure directed quantitative structure-activity relationships: 4—additional parameters for hydrophobic and dispersive interactions and their application for an automated superposition of certain naturally occurring nucleoside antibiotics. J. Chem. Inform. Comput. Sci..

[21] Veber DF (2002). Molecular properties that influence the oral bioavailability of drug candidates. J. Med. Chem..

[22] Yang H (2014). A novel systems pharmacology model for herbal medicine injection: a case using reduning injection. BMC Complem. Altern. Med..

[23] Liu X (2010). PharmMapper server: a web server for potential drug target identification using pharmacophore mapping approach. Nucleic Acids Res..

[24] Luo H (2011). DRAR-CPI: a server for identifying drug repositioning potential and adverse drug reactions via the chemical-protein interactome. Nucleic Acids Res..

[25] Wang X (2017). PharmMapper 2017 update: a web server for potential drug target identification with a comprehensive target pharmacophore database. Nucleic Acids Res..

[26] Amberger, J. S., Bocchini, C. A., Schiettecatte, F., Scott, A. F. & Hamosh, A. OMIM. org: online mendelian inheritance in man (OMIM®), an online catalog of human genes and genetic disorders. *Nucleic Acids Res.* **43**, D789–D798 (2015). 10.1093/nar/gku1205 PMC4383985 25428349

[27] Consortium, U (2019). UniProt: a worldwide hub of protein knowledge. Nucleic Acids Res..

[28] Warde-Farley D (2010). The GeneMANIA prediction server: biological network integration for gene prioritization and predicting gene function. Nucleic Acids Res..

[29] Storey JD, Tibshirani R (2003). Statistical significance for genomewide studies. Proc. Natl. Acad. Sci. USA.

[30] Jung K, Friede T, Beißbarth T (2011). Reporting FDR analogous confidence intervals for the log fold change of differentially expressed genes. BMC Bioinform..

[31] Benjamini Y, Hochberg Y (1995). Controlling the false discovery rate: a practical and powerful approach to multiple testing. J. R. Stat. Soc. Ser. B (Methodol.).

[32] Mostafavi S, Ray D, Warde-Farley D, Grouios C, Morris Q (2008). GeneMANIA: a real-time multiple association network integration algorithm for predicting gene function. Genome Biol..

[33] Chan JN, Nislow C, Emili A (2010). Recent advances and method development for drug target identification. Trends Pharmacol. Sci..

[34] Sherman BT, Lempicki RA (2009). Systematic and integrative analysis of large gene lists using DAVID bioinformatics resources. Nat. Protoc..

[35] Zhou, G. *et al.* NetworkAnalyst 3.0: a visual analytics platform for comprehensive gene expression profiling and meta-analysis. *Nucleic Acids Res.* **47**, W234–W241. 10.1093/nar/gkz240 (2019). 10.1093/nar/gkz240 PMC6602507 30931480

[36] Kuleshov MV (2016). Enrichr: a comprehensive gene set enrichment analysis web server 2016 update. Nucleic Acids Res..

[37] Nebert DW, Russell DW (2002). Clinical importance of the cytochromes P450. Lancet.

[38] Manikandan P, Nagini S (2018). Cytochrome P450 structure, function and clinical significance: a review. Curr. Drug Targets..

[39] Kang YJ, Park KK, Chung WY, Hwang JK, Lee SK (2009). Xanthorrhizol, a natural sesquiterpenoid, induces apoptosis and growth arrest in HCT116 human colon cancer cells. J. Pharmacol. Sci..

[40] Joo MK (2010). W1740 anti-cancer effects of xanthorrhizol and astaxanthine in esophageal cancer cell lines. Gastroenterology.

[41] Cheah YH (2009). Combined xanthorrhizol-curcumin exhibits synergistic growth inhibitory activity via apoptosis induction in human breast cancer cells MDA-MB-231. Cancer Cell Int..

[42] Beebe, M. *et al.* Synergistic impact of xanthorrhizol and d-δ-tocotrienol on the proliferation of murine B16 melanoma cells and human DU145 prostate carcinoma cells (P06–042–19). *Curr. Dev. Nutr.* **3**, P006–042–019 (2019). 10.1080/01635581.2020.1807573 32811212

[43] Consortium, G. O (2019). The gene ontology resource: 20 years and still GOing strong. Nucleic Acids Res..

[44] Hwang J-K, Shim J-S, Baek N-I, Pyun Y-R (2000). Xanthorrhizol: a potential antibacterial agent from *Curcuma xanthorrhiza* against *Streptococcus mutans*. Planta Med..

[45] Lee, S. K. *et al.* Suppressive effect of natural sesquiterpenoids on inducible cyclooxygenase (COX-2) and nitric oxide synthase (iNOS) activity in mouse macrophage cells. *J. Environ. Pathol. Toxicol. Oncol.* **21** (2002). 12086400

[46] Lim CS (2005). Antioxidant and antiinflammatory activities of xanthorrhizol in hippocampal neurons and primary cultured microglia. J. Neurosci. Res..

[47] Kim, M.-B., Kim, C., Song, Y. & Hwang, J.-K. Antihyperglycemic and anti-inflammatory effects of standardized Curcuma xanthorrhiza Roxb. extract and its active compound xanthorrhizol in high-fat diet-induced obese mice. *Evid. Based Complement. Altern. Med.* (2014). 10.1155/2014/205915 10.1155/2014/205915 PMC4094874 25053966

[48] Kim SH, Hong KO, Chung W-Y, Hwang JK, Park K-K (2004). Abrogation of cisplatin-induced hepatotoxicity in mice by xanthorrhizol is related to its effect on the regulation of gene transcription. Toxicol. Appl. Pharmacol..

[49] Kang Y-J, Park K-K, Chung W-Y, Hwang J-K, Lee SK (2009). Xanthorrhizol, a natural sesquiterpenoid, induces apoptosis and growth arrest in HCT116 human colon cancer cells. J. Pharmacol. Sci..

[50] Choi M-A, Kim SH, Chung W-Y, Hwang J-K, Park K-K (2004). Xanthorrhizol, a natural sesquiterpenoid from Curcuma xanthorrhiza, has an anti-metastatic potential in experimental mouse lung metastasis model. Biochem. Biophys. Res. Commun..

[51] Kim S (2019). PubChem 2019 update: improved access to chemical data. Nucleic Acids Res..

[52] Shannon P (2003). Cytoscape: a software environment for integrated models of biomolecular interaction networks. Genome Res..

[53] Kanehisa M, Goto S (2000). KEGG: kyoto encyclopedia of genes and genomes. Nucleic Acids Res..

[54] Szklarczyk D (2015). STRING v10: protein–protein interaction networks, integrated over the tree of life. Nucleic Acids Res..

[55] Su G, Morris JH, Demchak B, Bader GD (2014). Biological network exploration with Cytoscape 3. Curr. Protoc. Bioinform..

[56] Berman HM (2000). The protein data bank. Nucleic Acids Res..

[57] Kulkarni SA (2020). Computational evaluation of major components from plant essential oils as potent inhibitors of SARS-CoV-2 spike protein. J. Mol. Struct..

[58] BIOVIA, D. S. Discovery studio modeling environment, release 2017, San Diego: DassaultSystèmes, 2016. Accessed 1 September 2016.

